# Effects of incorporating magnesium oxide and zirconium dioxide nanoparticles in acrylic resin denture base material: a comparative study

**DOI:** 10.3389/fdmed.2025.1667644

**Published:** 2025-10-07

**Authors:** Zena J. Wally, Ola M. Al-Jubouri, Zaid G. Al-Jlaihawi, Rajaa M. Almusawi, Rasha A. Alamoush, Julfikar Haider

**Affiliations:** ^1^ Department of Prosthodontic, Faculty of Dentistry, University of Kufa, Najaf, Iraq; ^2^ Department of Mechanical Engineering, Faculty of Engineering, University of Kufa, Najaf, Iraq; ^3^ Department of Fixed and Removable Prosthodontics, School of Dentistry, The University of Jordan, Amman, Jordan; ^4^ Department of Engineering, Manchester Metropolitan University, Manchester, United Kingdom

**Keywords:** biomaterials, heat cure resin, denture base, MgO nanoparticles, ZrO_2_ nanoparticles, density, surface roughness, hardness

## Abstract

**Objectives:**

The current study aimed to evaluate the effects of incorporating magnesium oxide (MgO) and zirconium oxide (ZrO_2_) nanoparticles individually or in combination into heat-cured acrylic resin on the polymer structure, density, surface roughness, hardness and flexural strength for denture base applications.

**Materials and methods:**

A total of 280 samples were produced and divided into seven groups (*n* = 10) according to the percentage of nanomaterial used: pure heat-cured samples (control group) and heat-cured samples containing nanoparticles at 0.5 wt.% MgO, 1 wt.% MgO, 0.5 wt.% ZrO_2_, 1 wt.% ZrO_2_, 0.5 wt.% MgO-ZrO_2_, and 1 wt.% MgO-ZrO_2_. Polymer chemistry, density, surface roughness, hardness and flexural strength of the tested groups were determined using Fourier transform infrared (FTIR) spectroscopy, the Archimedes method, a profilometer, a microhardness test and a universal testing machine respectively.

**Results:**

The results indicated that both the MgO and ZrO_2_ nanoparticles interacted primarily with the carbonyl oxygen atoms, leading to the formation of coordination and ionic bonds. This interaction enhanced crosslinking within the polymer matrix. Additionally, the density of the heat-cured acrylic resin increased in a dose-dependent manner when the nanoparticles were incorporated. The surface roughness decreased with increasing nanoparticle concentration, and the smoothest samples were reported with 1% MgO-ZrO_2_. The modified group, which included 0.5% MgO-ZrO_2_, exhibited the greatest surface hardness. However, compared with those of the control group, the hardness values notably decreased as the concentration of nanoparticles increased. This reinforcement also significantly improved the flexural strength when the amount of nanoparticles increased.

**Conclusions:**

MgO and ZrO₂ nanoparticles strengthen the polymer matrix by forming coordination bonds with carbonyl oxygens, increasing crosslinking. This increased the density and flexural strength while reducing the surface roughness but lowered the hardness at elevated concentrations. These findings suggest the potential for tailored polymer applications, improved denture performance, increased patient comfort, and longer-lasting dental prostheses.

## 1 Introduction

Despite the growing use of dental implants, traditional partial and complete dentures are frequently the preferred treatment for patients who have lost their teeth for economic and medical reasons (1). Polymethylmethacrylate (PMMA) is the preferred material for denture bases because of its favorable properties, such as biocompatibility, satisfactory aesthetics, ease of processing, affordability, and stability in the patient's mouth (2). PMMA is also used in various dental applications, including the fabrication of removable orthodontic devices and retainers, artificial teeth, and fixing broken or damaged dentures (3). Despite these advantages, inherent limitations often obstruct their clinical performance, such as suboptimal surface hardness, susceptibility to porosity, surface roughness, incomplete polymerization and lower fracture resistance. Approximately 68% of complete dentures are likely to fracture during the first 3 years of use because of the occlusal load or falling of the denture (4).

The incorporation of filler materials into denture base materials has emerged as a promising strategy to address these challenges. Several trials have been conducted to overcome the limitations of PMMA and expand its biomechanical properties and clinical application. Different materials at the micro- and nanoscale, such as fibers, fillers, metal wires or plates (5), hydroxyapatite and multiwalled carbon nanotubes (6), Ag-Zn zeolite (7), titanium dioxide (TiO_2_) (8), aluminum oxide (Al_2_O_3_) nanoceramics (9), silica (SiO_2_), zirconia (ZrO_2_) (10) and magnesium oxide (MgO) (11), are integrated into the matrix of PMMA. Previous studies have shown that the properties of PMMA nanocomposites are influenced by the type, shape, size and concentration of the nanofiller (1).

Compared with larger particles, nanoparticles (NPs) are notable for their tiny size, large surface area, and intense interaction with resin materials, which provide them with unique mechanical, chemical, electrical, optical, and magnetic properties (12). Metal oxides are among the various kinds of NPs and they are helpful because of their antibacterial qualities and the variety of their physical and chemical characteristics (13). MgO and ZrO_2_ are two promising filler nanomaterials with substantial potential for improving the properties of polymeric materials. Both types of nanoparticles are ceramic materials with many advantages, such as biocompatibility, significant strength and acceptance in terms of aesthetics, in contrast to other metal oxide NPs, making them ideal candidates for dental applications (12). Several studies have demonstrated the efficacy of MgO NPs in reducing and controlling plaque biofilms, enhancing the antibacterial properties of dental materials, and repairing primary tooth caries (14, 15). Moreover, MgO NPs have antiseptic properties against many oral microbes, such as the cariogenic bacteria *S. mutans* (16). The MgO nanoparticle-modified glass-ionomer cement showed antibacterial and antibiofilm activity toward cariogenic microorganisms (17).

In a few studies, MgO has shown exceptional antimicrobial properties and mechanical reinforcement capabilities. Acrylic resins containing 2%, 4%, or 6% MgO NPs or AgNPs showed remarkable antifungal activity against *Candida albicans* denture stomatitis (18). In another study by Wady et al., PMMA reinforced with MgO (NPs) was found to be less active in reducing *Candida albicans* than Ag (NPs) were (19). A study reported that incorporating MgO NPs into cold-cured acrylic resin materials enhanced the flexural strength at low concentrations (20). A study reported that incorporating a limited concentration of MgO (2% or 4%) increased the hardness of acrylic resin materials. However, increasing the concentration beyond this level adversely affects the mechanical properties and stability of the color (21). It has also been reported that adding 3% MgO (NPs) to silicone denture liners has a negligible effect on surface hardness but significantly enhances tensile bond strength (22).

ZrO_2_ NPs have shown to have antimicrobial properties in different studies and to strengthen performance of PMMA (23). The incorporation of ZrO_2_ into autopolymerized acrylic resin denture base effectively inhibited Candida adhesion (24). A composite of PMMA-ZrO_2_ integrated with MgO (NPs) recently showed excellent biocompatibility and anti-toxicity. No generation of reactive oxygen species (ROS) was detected in the gut of epithelial cells upon administration of different nanoparticle concentrations (2 µg/ml, 5 µg/ml and 10 µg/ml) (25). Moreover, TiO_2_/3D-printed PMMA composite showed cellular viability with no cytotoxic effect against human gingival fibroblast cells after 10 days. However, higher concentration (0.75 wt.%) showed a slight reduction in the antifungal activity against Candida albicans and confirming the material's biocompatibility (26). The strength enhancement was dependent on the ZrO_2_ concentration (27). The incorporation of different concentrations (2.5 wt.%, 5 wt.%, and 7.5 wt.%) of ZrO_2_ NPs into autopolymerized acrylic resin powder has been reported in the literature. The highest flexural strength was shown in the tested group treated with 7.5% ZrO_2_ NPs, whereas a higher impact strength was shown with 2.5% ZrO_2_NPs (27). In another study, ZrO_2_, Al_2_O_3_ and TiO_2_ particles were mixed with heat-cured acrylic resins and shown to increase the impact strength and fracture toughness and reduce water sorption and solubility (23). The incorporation of MgO at various concentrations into PMMA-ZrO_2_ hybrid composites was reported in a study by Kumari et al. (2024), and these particles effectively improved the density, compressive strength, Young's modulus, fracture toughness, and flexural properties of the composite (25). However, loading higher concentrations may cause agglomeration, producing localized stress points and unsettling the material's consistency (7).

Surface hardness is a key property that directly affects the wear resistance and scratch resistance of acrylic resins (28, 29). In clinical applications, materials with inadequate surface hardness are prone to abrasions, leading to microbial adhesion, biofilm formation, and, ultimately, decreased functionality and patient satisfaction (30). Likewise, porosity, which frequently results from insufficient polymerization or inappropriate processing, adversely affects a material's mechanical strength and aesthetic appearance (31). The presence of porosity allows the absorption of oral fluids, which can cause staining, bad smells, and a decrease in resin biocompatibility (32, 33). Surface roughness, another crucial parameter, impacts both the ability of a material to resist plaque accumulation and the degree of comfort experienced by patients (34). The overall performance of PMMA is closely related to the degree of polymerization. The residual monomer content from incomplete polymerization weakens the mechanical characteristics and increases the degree of cytotoxicity risk (35).

Both MgO and ZrO₂ NPs have been studied individually in various polymeric systems and have shown distinct benefits. An appropriate amount of MgO could improve the flexural strength and hardness, improving the quality of the denture bases (21). Nano-ZrO₂ showed particle size-dependent reinforcement of the PMMA composites (10). To the best of the authors' knowledge, only one study has explored the effects of the combination of high concentrations of ZrO₂ (5% wt.%) and MgO (2–6 wt.%) on the density, compressive strength, Young's modulus, fracture toughness, and flexural properties of PMMA with high wear volume at high concentrations (25). However, their study lacked a systematic assessment of the effects of adding ultralow concentrations (0.5–1.0 wt.%) of ZrO_2_ and MgO independently and their combinations on denture base material characteristics.

This study aims to produce an optimized MgO-ZrO₂ nanoparticle-incorporated resin composite for use as a denture base at ultralow concentrations. Here, we uniquely assessed their individual properties by incorporating precisely controlled and low loading concentrations (0.5–1 wt.% MgO, 0.5–1 wt.% ZrO₂) while also revealing their synergistic potential (0.5–1 wt.% combinations) when combined with heat-cured acrylic resins to determine their impact on properties that directly address denture longevity and patient comfort: (1) polymer functional groups, (2) density, (3) surface roughness, (4) hardness, and (5) flexural strength of heat-cured acrylic resins. This comprehensive approach has the potential not only to identify optimal nanoparticle loadings but also to reveal new structure‒property relationships for developing high-performance denture base materials.

Despite the growing interest in nanoparticle-reinforced PMMA, current studies on dental resin composites have yet to optimize the minimal concentration of NPs loaded in PMMA, as high additives have been shown to generate unfavorable trade-offs between wear and other mechanical properties. New approaches are required to limit this gap and achieve a balanced improvement through all necessary properties without negotiating biocompatibility. The novelty of our study is the exploration of a new strategy by investigating whether individual effects or a synergistic combination of MgO and ZrO₂ at ultralow doses could balance the improvement in most key properties at the same time without trade-offs.

The hypothesis of this study was that the effects of the addition of different NPs (MgO, ZrO_2_ and a combination of MgO-ZrO_2_) at different concentrations (0.5 wt.% and 1.0 wt.%) on the polymerization process, density, surface roughness, flexural strength and hardness of heat-polymerized PMMA would be significant. It was expected that the NPs would change the polymer chemistry and improve the physical and mechanical properties compared with those of the control.

## 2 Materials and methods

### 2.1 Specimen preparation and grouping

**Figure 1:**
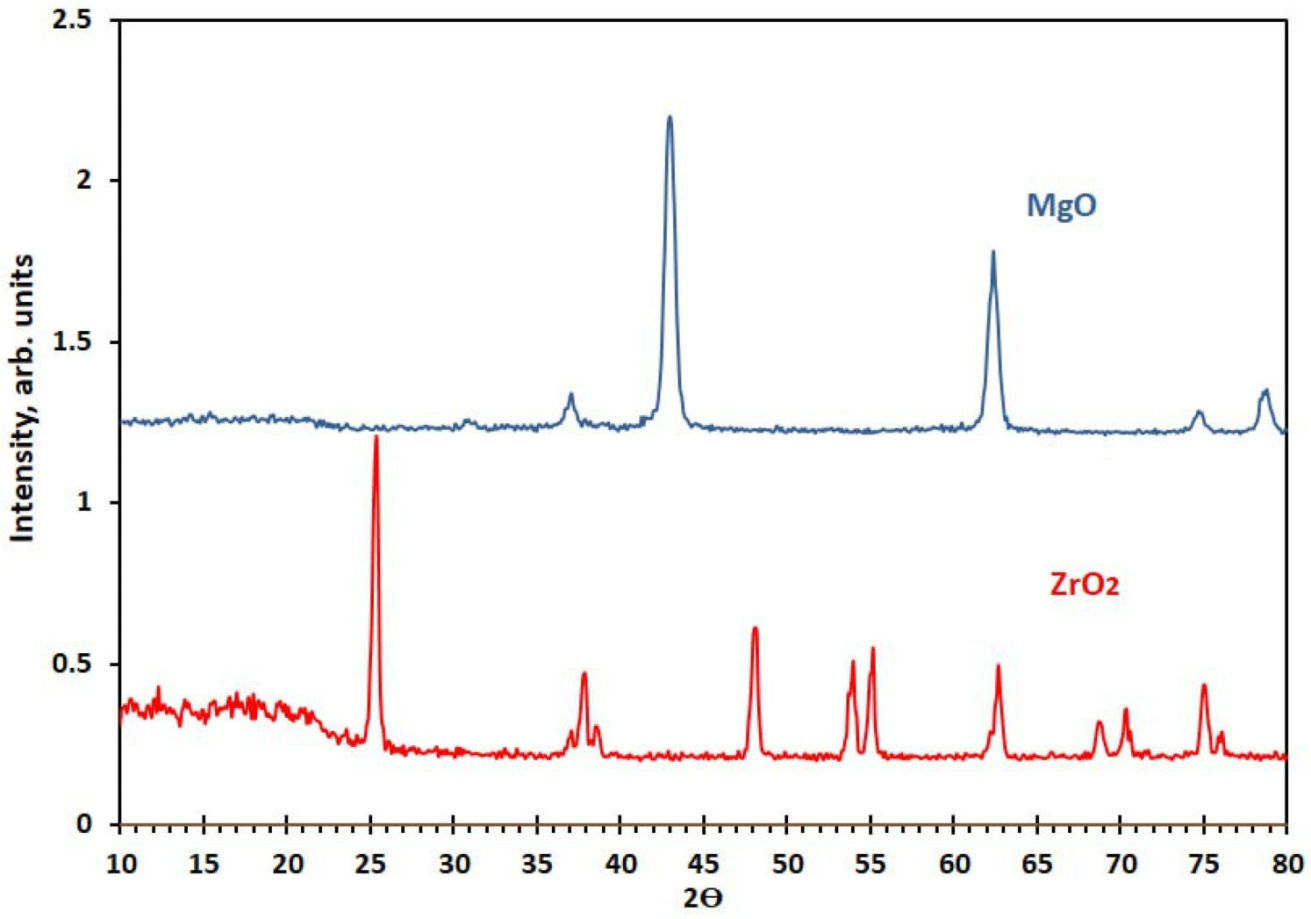
XRD analysis of the MgO and ZrO₂ nanoparticles used.

To examine and compare the effects of MgO and ZrO_2_ NPs on acrylic denture-based properties. In total, 280 samples were produced and allocated into 7 different groups: a control group without NPs and 6 experimental groups with nanoparticle variants (0.5 wt.% MgO, 1 wt.% MgO, 0.5 wt.% ZrO_2_, 1 wt.% ZrO_2_, 0.5 wt.% MgO-ZrO_2_, and 1 wt.% MgO-ZrO_2_). The impacts of these concentrations were evaluated for key material properties (density, surface roughness, hardness and flexural strength) of hot-cured acrylic resin. A sample size of *n* = 10 per group was used based on ISO 4049 recommendations for resin-based materials. This finding aligns with related studies assessing nanoparticle-enhanced dental resins (35, 36). The tested MgO and ZrO₂ NPs had a purity of 99.94%, as provided by the manufacturer (Changsha Santech Materials Company, China). The crystallite sizes of the MgO and ZrO_2_ powders were determined via x-ray diffraction (XRD) via an AL-2700 series diffractometer (Dandong Aolong Radiative Instrument Group Co., Ltd.). A Cu K*α* radiation source (*λ* = 0.15418 nm) was used for the measurements, which were performed in the 2*θ* range of 10–80° at a scan rate of 3° min^−1^. The mean crystallite dimensions were calculated via the Scherrer equation:

$$D=\frac{K\lambda }{\beta_{s}\cos\;\theta }$$

where *D* is the crystallite size (nm), *K* = 0.9 (Scherrer constant), *λ* denotes the x-ray wavelength, *β*_*s* represents the full width at half maximum (FWHM) of the most intense diffraction peaks, and *θ* is the Bragg angle. On the basis of this analysis, the average crystallite sizes were determined to be 17.7 ± 2.9 nm for MgO and 26.1 ± 4.9 for ZrO2, as presented in Figure 1.

A commercially available heat-curing acrylic resin (Superacryl™ Plus, Sofa Dental; Spofa Dental, Czech Republic) composed of powder and liquid was used to fabricate acrylic samples. Different weight percentages of MgO and ZrO_2_, separately or in combination, were incorporated with the heat-cured acrylic resin: MgO at 0.5 and 1, ZrO_2_ at 0.5 and 1, and MgO-ZrO_2_ at 0.5 and 1. The concentrations were selected on the basis of a preliminary investigation, which revealed that a high concentration greatly reduced most of the mechanical properties. The required weight of the powder of the polymer and NPs was determined via an electronic balance (Sartorius, Germany, with an accuracy of 0.0001 g) for each group. The details of the nanoparticle weight percentages used in this study are listed in Table 1.

**Table 1:** Weight percentages (wt.%) of MgO and ZrO_2_ in combination with heat-cured acrylic resin in the control and experimental groups used in the current study.

Group ID	Group name	MgO and ZrO_2_ NPs weight (g)	Heat-cured acrylic resin powder (g)	Heat-cured acrylic resin monomer (ml)
A	Control (Pure PMMA)	0.00	100.00	50
B	0.5 MgO	0.50 MgO	99.50	50
C	1 MgO	1.00 MgO	99.00	50
D	0.5 ZrO_2_	0.50 ZrO_2_	99.50	50
E	1 ZrO_2_	1.00 ZrO_2_	99.00	50
F	0.5 MgO-ZrO_2_	0.25 MgO + 0.25 ZrO_2_	99.50	50
G	1 MgO-ZrO_2_	0.50 MgO + 0.50 ZrO_2_	99.50	50

In accordance with the ISO 20795-1:2013 standard for denture base polymers, rectangular plastic molds with dimensions of 50 mm × 20 mm × 2.5 mm (length, width and thickness) were used for hardness testing (Vickers method), and 80 mm × 10 mm × 2.5 mm were used for surface roughness testing.

The plastic molds were individually invested to approximately one half of their depth into type III dental stone (type 3 ELITE Model, Zhermack, Italy) in the lower part of a traditional dental flask (Brass flask, Kavo EWL, Germany). After stone setting, the molds were coated with a separating medium (Cold Mold Seal, Pyrax, India), which was left to dry for 15–20 min. To prevent air entrapment, the upper part of the flask was then adjusted and filled with another stone layer under a vibrator (Vibromaster No. 24122; Bego Bremer Goldschagerel Co., Bremen, Germany) at 3,000 cycles/min (low) and 6,000 cycles/min (high) frequencies for 1 min. After the stone had set, the plastic patterns were carefully removed from the mold. Then, both halves of the flask were coated with a separating medium (cold mold seal) to prepare them for packing with acrylic dough. Direct mixing and ultrasonication were selected for this study due to their affordability, ease of implementation, and recognized efficiency in attaining uniform NPs spreading without compromising the mechanical or biocompatibility properties of PMMA (8). The proportion of acrylic resin used for mixing was 2:1 by a ratio (polymer/monomer) and mixing and manipulation were performed according to the manufacturer's recommendations.

To eliminate nanoparticle aggregation and produce a consistent resin matrix, they were added to the monomer, and then, this mixture was ultrasonically processed (Soniprep150, England) at 120 W and 60 kHz for two hours (8). The mixture of monomer and tested NPs was then instantly mixed manually with the polymer powder to eliminate possible aggregation. The mixing was carried out in a clean dry jar with a mixing spatula at room temperature (23 °C ± 5 °C) until the dough stage was reached in approximately 10 min, which was appropriate for manipulation. The dough was then immediately packed according to the stone index in the dental flask. The flasks were progressively compressed in a hydraulic press (8.5–9.0 MPa for 1 min) and then polymerized in a water bath (Elektro mag M 24 K Bacteriology, Germany) at 100 °C for 8 h.

The flask was left to cool at room temperature for one day to prevent any shrinkage. All acrylic samples were then deflasked and removed from the stone mold. The acrylic samples were removed from the flask and manually finished with constantly draining water using ever finer grades of silicon carbide paper (grades 120 to 40 µm). To ensure accurate measurements, the samples were prepared with a flat, smooth surface for hardness testing and a flat, polished surface for surface roughness testing. Every sample was finished and polished by using a bristle brush and pumice (Pumice Fine/Coarse; Kerr Dental, USA) with a lathe polishing machine (Baldor 340 Dental polishing lathes, USA). The samples were kept in distilled water at 37 °C for 7 days prior to testing in compliance with ADA specification NO.12 (1999).

### 2.2 Surface characterization and properties

#### 2.2.1 Scanning electron microscopy (SEM)

This study utilized scanning electron microscopy (SEM) (Inspect S50 manufactured by the FEI Company in the Netherlands) alongside energy dispersive x-ray spectroscopy (EDS) to examine the surface morphology of heat-cured acrylic resin samples. The observation process was conducted under low vacuum conditions (60 Pa) with a low accelerating voltage of 20 kV, a small spot size (3 nm), and a short imaging duration (30 s), eliminating the need for chemical fixation. To prepare the samples, they were mounted on two sides, and then the samples were coated with gold via sputter coating equipment. The morphologies of all the acrylic resin samples were subsequently evaluated and compared.

#### 2.2.2 Transmission electron microscope (TEM)

Transmission electron microscopy (TEM) (Zeiss-EM10C-100 KV, Germany) was used to evaluate the dispersion of the NPs in the cured resin samples.

#### 2.2.3 Fourier transform infrared (FTIR) spectral analysis

To evaluate the functional groups in the modified acrylic resin samples, Fourier transform infrared spectral analysis was conducted with a Digilab Excalibur FTS 3000 Mx spectrometer with a DTGS detector. This spectrometer consists of ZnSe-based attenuated total reflectance (ATR) using various reflection and transmission attachments. Potassium bromide (approximately 200 mg) was mixed with each sample (approximately 2 mg) via hydraulic pressure under 8,000 (kg) pressure in a special matrix. The transmission spectra of the samples were recorded at a resolution of 4 cm^–1^ in the 4,000–400 cm^–1^ range. The number of scans was determined on the basis of the level of the signal obtained within 32 scans. Finally, Merlin (FTS 3000) software was used to correct the obtained spectral baseline. This method involves measuring the absorption of infrared radiation by a polymer, providing information about functional groups. The degree of polymerization was estimated by analyzing the changes in specific peaks.

#### 2.2.4 Density

**Figure 2:**
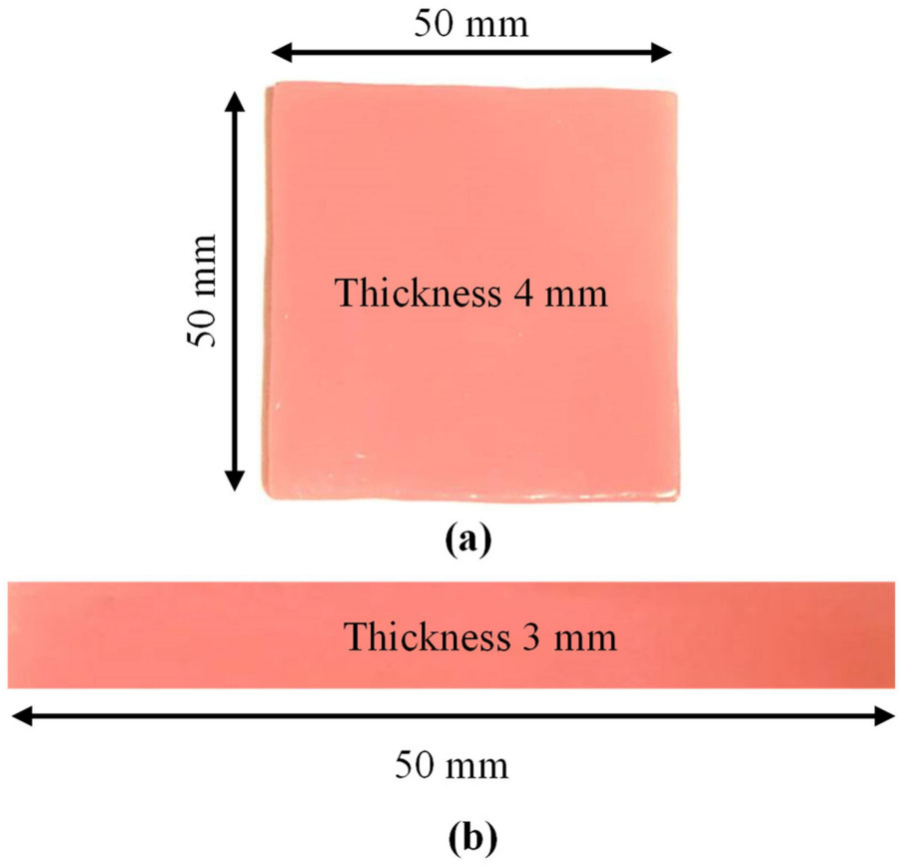
Acrylic samples used for **(a)** roughness and hardness and **(b)** density and flexural strength tests.

The theory of Archimedes was used to investigate the void volume within the material and density changes, with the mass (m) measured on a critical and precision density balance (Mettler Toledo, Switzerland) with 0.1 mg resolution and the volume (V) determined by the principle. Samples measuring 64 mm × 10 mm × 3.3 mm ± 0.2 were used for density tests according to ISO 20795-1:2013, as shown in Figure 2. The density of the sample (*ρ*) was calculated via the following formula (Equation 1):

(1) ρ=m/V

where *ρ* is the density [g/cm^3^], *m* is the mass [g] and *V* is the volume [cm^3^].

#### 2.2.5 Surface roughness

A roughness tester (HSR 210, Hensgrand, Jinan, China) was used to evaluate the surface roughness of the samples with a length of 2 mm. Prior to testing, the samples were immersed in distilled water at 37 °C for 48 h. To accurately measure the microgeometry of the surface, the samples were placed on a stable bench during the test. To trace the profile of the surface irregularities, measurements were taken at four different sites for each sample, and the mean values of these readings were calculated (7). Square-shaped samples with dimensions of 50 mm × 50 mm × 4 mm ± 0.2 mm were used for the surface roughness and hardness tests, according to ISO 20795-1:2013, as shown in Figure 2.

#### 2.2.6 Vickers hardness

A digital instrument micro hardness tester (Vickers, Germany) was utilized in this study to carry out the hardness measurements. The test was conducted with a load of 50 N, which is appropriate for acrylic resin materials. To confirm accurate measurements, the contact surface of the tester was aligned with the support platform of the sample.

During testing, the gap between the sample surface and the indenter was adjusted to 5–12 mm. The indenter remained in contact with the sample for six seconds. The measurements then possessed an intermediary from the scale, which is a number shown on the digital LCD screen. Five readings were taken from multiple areas of each sample, and the mean of these readings was calculated.

#### 2.2.7 Flexural strength

To calculate the flexural strength, a rectangular bar measuring 64 mm × 10 mm × 3.3 mm ± 0.2 was created in compliance with the ISO 20795-1: 2013 standard, as shown in Figure 2. This test was conducted on a universal testing system (model WDW-5E, China) using a three-point bend fixture. Before the tests were conducted, all the samples were incubated for ten days at room temperature in distilled water. The space between the specimen supports was 40 mm, and the samples were loaded until they fractured at a crosshead speed of 5 mm/min. The diameter of the loading plunger and supporting rollers was 20 mm. The greatest force applied on the samples was measured, and the flexural strength values were measured in MPa according to the following formula (Equation 2):

(2) $$F=3\mathrm{WL}/2bd^{2}$$

where *F* is the flexural strength (MPa), W is the load (*N*) at fracture, L is the distance between the supports (mm), b is the width of the sample (mm) and d is the specimen thickness (mm).

#### 2.2.8 Fracture behaviour analysis process

SEM was also used to analyze the fractured surface of the heat-cured acrylic resin samples. Two broken pieces (Fragments A and B) from each sample were made and visually examined via a claw-mounted magnifier (Western 9051 L; Western Ophthalmic Corp., São Paulo, Brazil) at ×4 magnification power (37).

### 2.3 Statistical analysis

To evaluate how MgO, ZrO_2_, and MgO-ZrO_2_ NPs influence the density, surface roughness, Vickers hardness and flexural strength of acrylic resin, statistical analyses were performed. The findings are presented as the means with standard deviations (SDs). The experimental data were compared against control values to determine any significant effects. GraphPad Prism 11 (Boston, MA, USA) was used to analyze the data via one-way ANOVA. *p* values less than 0.05 were regarded as statistically significant. A *post-hoc* power analysis by G* power analysis (Version 3.1) used to determine the sample size which confirmed the large effect sizes observed (*f* = 0.88–4.19) and the sample size *n* = 10 per group provided excellent statistical power above the conventional threshold of 0.80. This minimized the risk of Type II error to less than 0.5% for all measured properties. Additionally, the Shapiro‒Wilk test was used to evaluate the normal distribution of the data, whereas the Brown‒Forsythe test was employed to evaluate the homogeneity of variance.

## 3 Results

### 3.1 Material composition and particle dispersion

**Figure 3:**
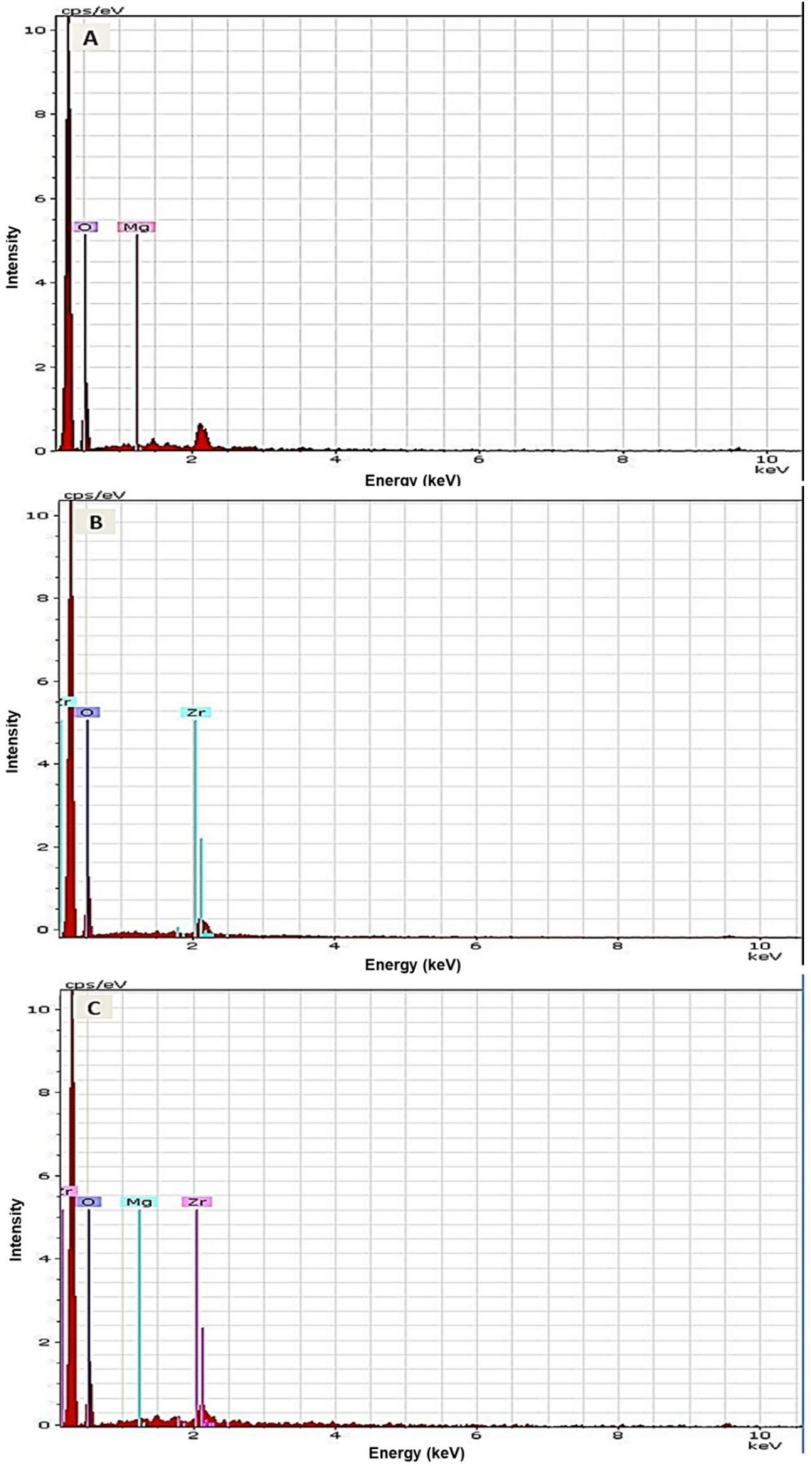
Representative EDX images of heat-cured acrylic resin incorporated with **(A)** MgO, **(B)** ZrO_2_ and **(C)** MgO-ZrO_2_ confirming the presence of NPs in the acrylic resin samples.

**Figure 4:**
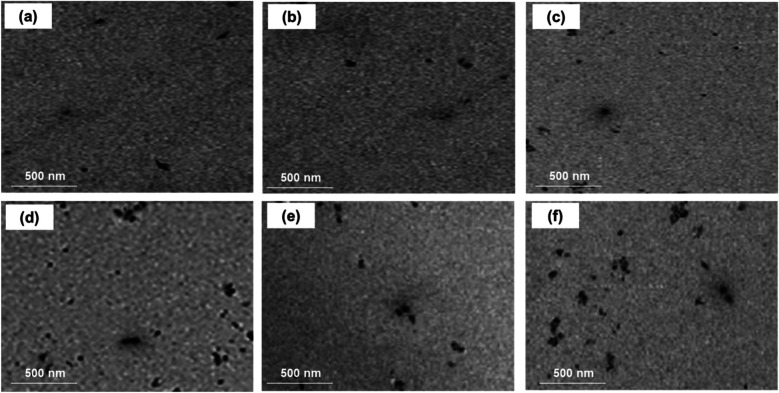
TEM images of heat-cured acrylic resin samples incorporated with **(a)** 0.5% MgO, **(b)** 0.5% ZrO_2_, **(c)** 0.5% MgO-ZrO_2_, **(d)** 1% MgO, **(e)** 1% ZrO_2_, and **(f)** 1% MgO-ZrO_2_ and **(c)** MgO-ZrO_2_ nanoparticles.

The results of elemental and structural analysis (EDX) of the polymerized acrylic resin loaded with MgO and ZrO_2_ and the MgO-ZrO_2_ NPs are displayed in Figure 3. The EDX spectrum revealed magnesium, zirconium and oxygen atoms in the tested samples, which confirmed the presence of these NPs within the produced heat–cured resin. Generally, the TEM images confirmed the good dispersion of the NPs within the cured resin samples of all the tested groups. However, agglomerates were noted at higher concentrations, as shown in Figure 4.

### 3.2 Fourier transform infrared (FTIR) spectral analysis

**Figure 5:**
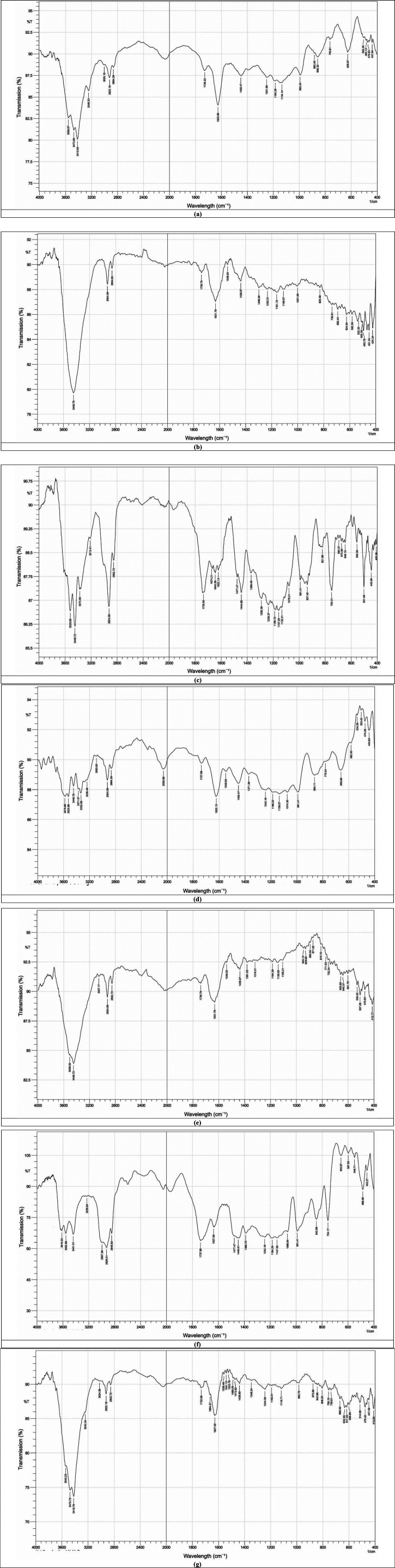
FTIR spectra of pure acrylic and acrylic with ZrO_2_ and MgO nanoparticles: **(a)** control, **(b)** heat-cured acrylic+0.5% ZrO_2_, **(c)** Heat-cured acrylic+1.0% ZrO_2_, **(d)** Heat-cured acrylic+0.5% MgO, **(e)** Heat-cured acrylic+1.0% MgO, **(f)** Heat -cured acrylic+0.5% MgO-ZrO_2_, **(g)** Heat -cured acrylic+1.0% MgO-ZrO_2._

Generally, the FTIR analysis confirmed that the presence of MgO and ZrO₂ NPs altered the chemical environment of the acrylic polymer. The FTIR spectra of acrylic resin with ZrO_2_ and MgO presented in Figure 5 show the main absorption bands formed for the selected materials. The spectrum of the control group could be compared with those of the nanocomposite groups reinforced with ZrO_2_ and MgO NPs at approximately 1,450–1,728 cm^−1^ and 2,864–2,922 cm^−1^, which were related to the asymmetric tension of C–H flexion and the C–H bonds in the methyl groups, respectively. In addition, Figure 5a shows many bands ranging from 626 cm^−1^–993 cm^−1^, which are related to hydroxyl O–H flexion and C–O stretching modes. The characteristic peaks in the pure acrylic spectrum were detected at 1,626 cm^−^^1^ (C = O stretching of the ester carbonyl group), 2,950–2,850 cm^−1^ (C-H stretching vibrations), and 1,150–1,250 cm^−1^ (C-O stretching vibrations), which are typical peaks for acrylic polymers.

Upon the addition of 1.0% Zr, as shown in Figure 5c, the band corresponding to the C = O stretching peak slightly shifted to 1,735 ± 1.9 cm^−^^1^ (SD, *n* = 3). New peaks ranging from 1,600 ± 2.5–1,675 ± 2.2 cm^−^^1^ appear, which are attributed to Zr–O bonds, further indicating the chemical interaction of Zr with the acrylic matrix.

In parallel, the addition of Mg led to a wide and stronger shift in the C = O peak to 1,739 ± 1.7 cm^−^^1^(SD, *n* = 3), in addition to the release of a new band peak at 1,631 ± 1.1 cm^−^^1^ (SD, *n* = 3), assigned to Mg‒O vibration bonds, indicating the potential for stronger ionic or coordination interactions than those of Zr. However, the FTIR spectrum of the Zr and Mg incorporated into the acrylic material shows a synergistic effect via the C = O peak shifting further to 1,732 ± 1.8 cm^−^^1^ (SD, *n* = 3) and the simultaneous presence of both Zr-O (1,628 ± 2.9 cm^−^^1^) and Mg-O (1,650 ± 1.7 cm^−^^1^) peaks. This could indicate that Zr and Mg interact essentially with the carbonyl oxygen atoms, which in turn leads to the formation of coordination and ionic bonds, reinforcing crosslinking polymer confinement effects within the polymer matrix.

### 3.3 Density measurement

**Figure 6:**
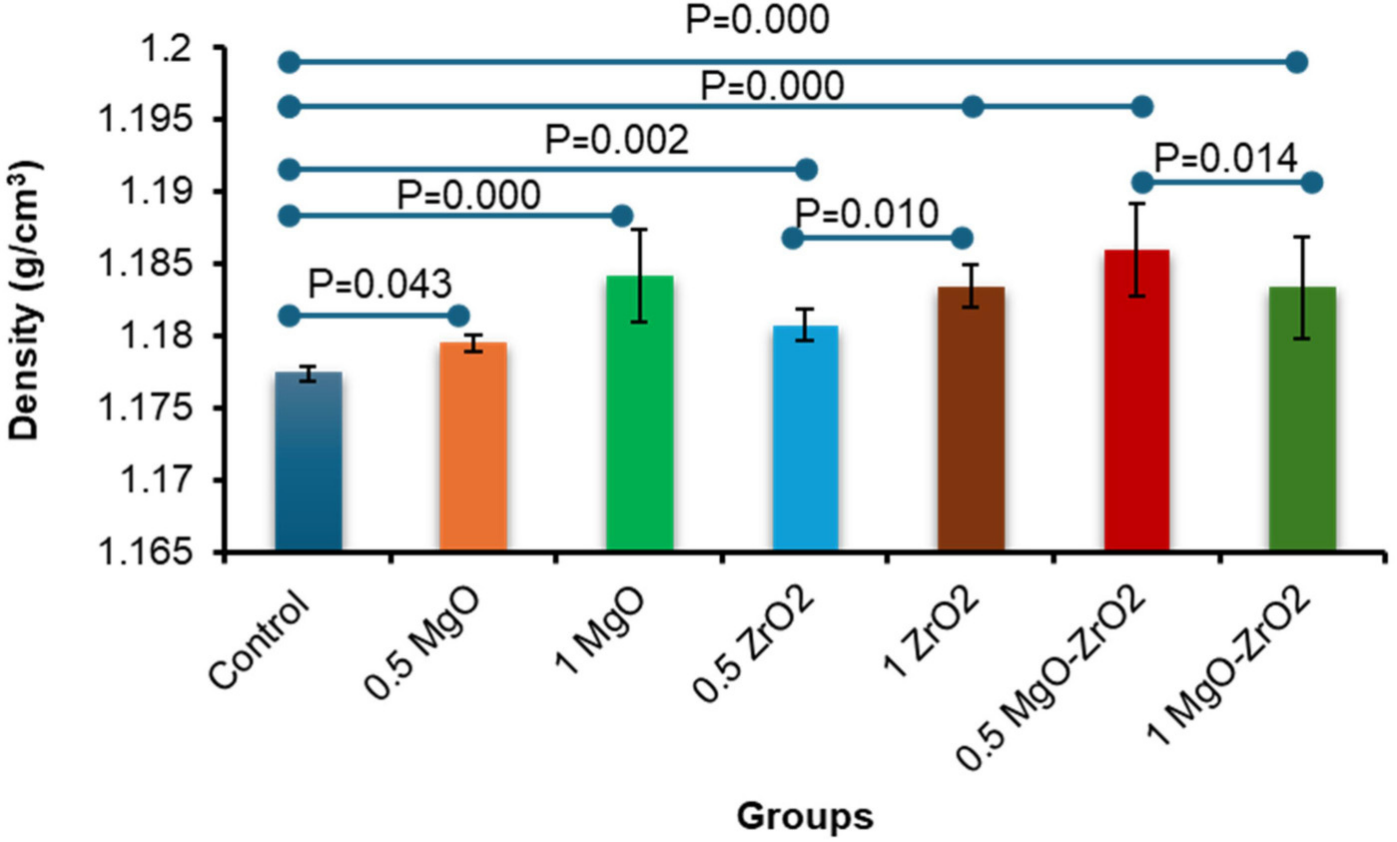
Bar chart of the density of heat-cured acrylic incorporated with 0.5% MgO, 1% MgO, 0.5% ZrO_2_, 1% ZrO_2_, 0.5% MgO-ZrO_2_ and 1% MgO-ZrO_2_ NPs and the control. All significant relationships cannot be shown in the figure due to limited space. Only control with all other groups and same NPs or combination NPs groups with different concentrations are shown. See Table A1 in Appendix for the additional statistical analysis.

In general, the density of heat-cured acrylic resin significantly increased with the addition of MgO and ZrO_2_ NPs. The highest mean density value (1.1859) was reported for the 0.5 MgO-ZrO_2_ group, whereas the control group presented the lowest density value (1.1774). Interestingly, higher concentrations of MgO and ZrO_2_ and the combination of MgO-ZrO_2_ NPs with acrylic resin led to a notable improvement in density, as demonstrated in Table 2 and Figure 6.

**Table 2:** Descriptive statistics for the density of heat-cured acrylic resin incorporated with 0.5% MgO, (C) 1% MgO, 0.5% ZrO_2_, 1% ZrO_2_, 0.5% MgO-ZrO_2_, 1% MgO-ZrO_2_ and the control.

Groups	*N*	Mean (g/cm^3^)	Std. deviation	Minimum	Maximum
Control	10	1.1774	0.0005	1.1768	1.1778
0.5 MgO	10	1.1795	0.0006	1.1782	1.1799
1.0 MgO	10	1.1841	0.0032	1.175	1.1855
0.5 ZrO_2_	10	1.1807	0.0011	1.1799	1.1822
1.0 ZrO_2_	10	1.1834	0.0015	1.1811	1.185
0.5 MgO-ZrO_2_	10	1.1859	0.0032	1.1841	1.1951
1.0 MgO-ZrO_2_	10	1.1834	0.0035	1.1778	1.1868

### 3.4 Surface roughness

**Figure 7:**
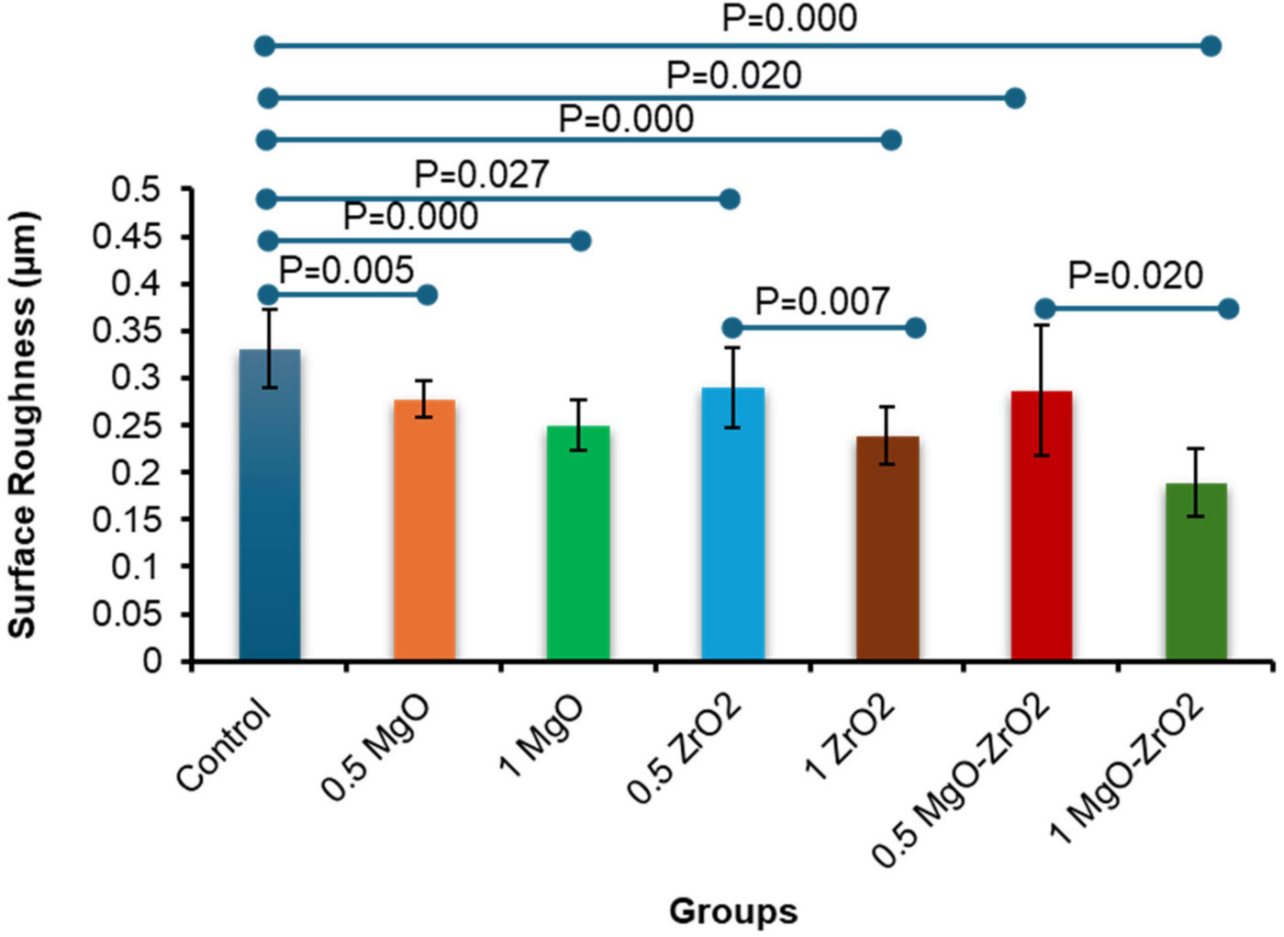
Bar chart of the surface roughness of heat-cured acrylic samples containing 0.5% MgO, 1% MgO, 0.5% ZrO_2_, 1% ZrO_2_, 0.5% MgO-ZrO_2_ and 1% MgO-ZrO_2_ NPs and the control. All significant relationships cannot be shown in the figure due to limited space. Only control with all other groups and same NPs or combination NPs groups with different concentrations are shown. See Table A1 in Appendix for the additional statistical analysis.

As presented in Table 3 and Figure 7, the integration of MgO-ZrO_2_ into the heat-cured acrylic resin at dissimilar concentrations markedly reduced the surface roughness counts compared with those of the control group. The maximum surface roughness amount (0. 331 ± 0.041) was recorded in the control group. In comparison, the lowest count (0.189 ± 0.035) was recorded for the heat-cured resin containing 1 MgO-ZrO_2_. The values of surface roughness decreased in a dose-dependent manner as the nanoparticle percentage increased, and the smoothness increased. However, there was no notable difference between MgO and ZrO_2_ at most concentrations used.

**Table 3:** Descriptive statistics for the surface roughness of heat-cured acrylic resin incorporated with 0.5% MgO, (C) 1% MgO, 0.5% ZrO_2_, 1% ZrO_2_, 0.5% MgO-ZrO_2_, 1% MgO-ZrO_2_ and the control.

Groups	*N*	Mean (µm)	Std. deviation	Minimum	Maximum
Control	10	0.331	0.041	0.284	0.374
0.5 MgO	10	0.278	0.020	0.263	0.301
1 MgO	10	0.251	0.027	0.219	0.272
0.5 ZrO_2_	10	0.290	0.042	0.252	0.347
1 ZrO_2_	10	0.240	0.030	0.214	0.283
0.5 MgO-ZrO_2_	10	0.287	0.069	0.201	0.371
1 MgO-ZrO_2_	10	0.189	0.035	0.143	0.224

### 3.5 Vickers hardness

**Figure 8:**
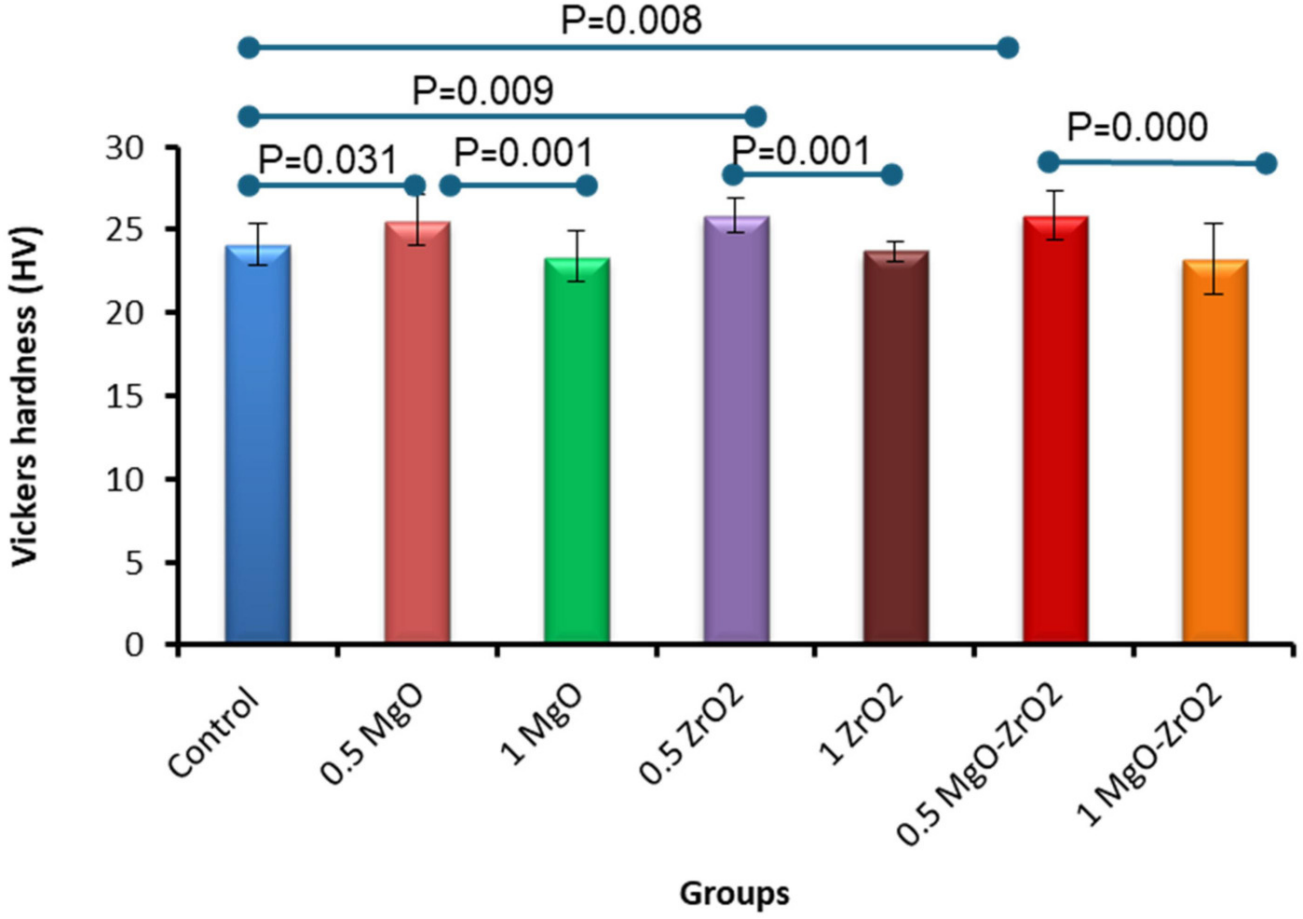
Bar chart of the vickers hardness of heat-cured acrylic samples containing 0.5% MgO, 1% MgO, 0.5% ZrO_2_, 1% ZrO_2_, 0.5% MgO-ZrO_2_ and 1% MgO-ZrO_2_ NPs and the control. All significant relationships cannot be shown in the figure due to limited space. Only control with all other groups and same NPs or combination NPs groups with different concentrations are shown. See Table A1 in Appendix for the additional statistical analysis.

Overall, the integration of heat-cured PMMA with 0.5% MgO or ZrO_2_ NPs alone or in combination significantly increased the surface hardness compared with that of the control group, as shown in Table 4 and Figure 8. The modified group incorporated with 0.5% MgO-ZrO_2_ had the highest surface hardness value of 25.89 ± 1.49, whereas the 1% MgO-ZrO_2_ group had the lowest value of 23.25 ± 2.13. Interestingly, as the concentration of NPs increased, the hardness of the heat-cured acrylic resin samples decreased. Furthermore, this reduction was not significant compared with that of the pure resin.

**Table 4:** Descriptive statistics for the vickers hardness of heat-cured acrylic resin incorporated with 0.5% MgO, (C) 1% MgO, 0.5% ZrO_2_, 1% ZrO_2_, 0.5% MgO-ZrO_2_, 1% MgO-ZrO_2_ and the control.

Groups	*N*	Mean (HV)	Std. deviation	Minimum	Maximum
Control	10	24.15	1.26	22.30	25.75
0.5 MgO	10	25.56	1.52	22.80	27.70
1 MgO	10	23.38	1.55	20.08	25.48
0.5 ZrO_2_	10	25.87	1.07	24.27	27.27
1 ZrO_2_	10	23.72	0.59	22.91	24.91
0.5 MgO-ZrO_2_	10	25.89	1.49	24.19	29.29
1 MgO-ZrO_2_	10	23.25	2.13	20.75	27.75

### 3.6 Flexural strength

**Figure 9:**
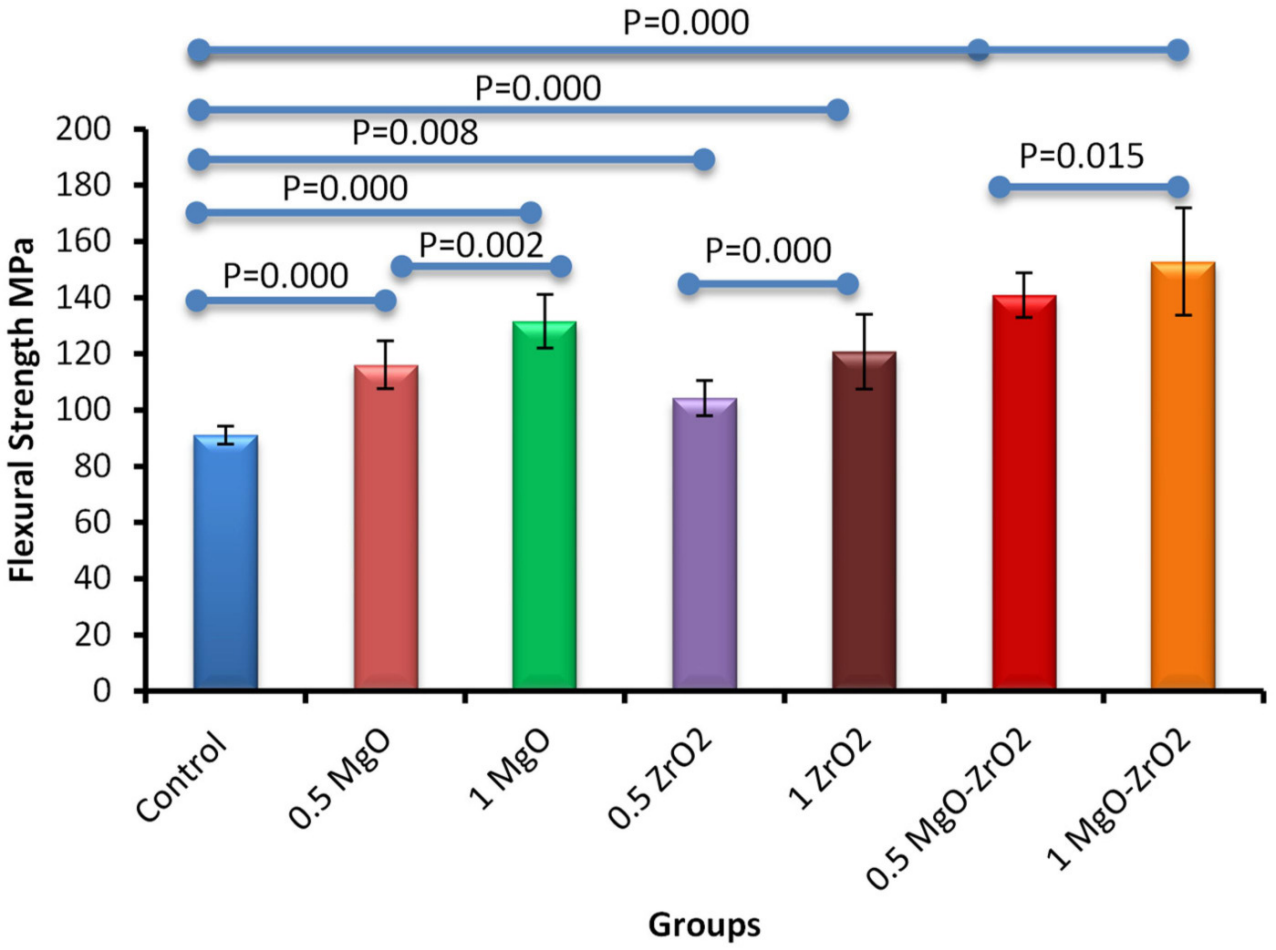
Bar chart of the flexural strength of heat-cured acrylic samples containing 0.5% MgO, 1% MgO, 0.5% ZrO_2_, 1% ZrO_2_, 0.5% MgO-ZrO_2_ and 1% MgO-ZrO_2_ NPs and the control. All significant relationships cannot be shown in the figure due to limited space. Only control with all other groups and same NPs or combination NPs groups with different concentrations are shown. See Table A1 in Appendix for the additional statistical analysis.

In general, the incorporation of heat-cured acrylic resin with MgO and ZrO_2_ NPs alone or in combination at various concentrations significantly improved the flexural strength values compared with those of the control group, as shown in Figure 9 and Table 5. The modified group incorporated with 1% MgO-ZrO_2_ had the highest flexural strength value of 152.85 ± 19.03, whereas the control group had the lowest value of 91.15 ± 3.22. Interestingly, the flexural strength of the samples improved as the proportion of NPs increased.

**Table 5:** Descriptive statistics for the flexural strength of heat-cured acrylic resin incorporated with 0.5% MgO, (C) 1% MgO, 0.5% ZrO_2_, 1% ZrO_2_, 0.5% MgO-ZrO_2_, 1% MgO-ZrO_2_ and the control.

Groups	*N*	Mean (MPa)	Std. deviation	Minimum	Maximum
Control	10	91.15	3.22	86.45	95.33
0.5 MgO	10	116.08	8.51	99.45	129.56
1 MgO	10	131.61	9.55	120.25	151.35
0.5 ZrO_2_	10	104.30	6.27	97.42	112.69
1 ZrO_2_	10	120.78	13.33	99.01	140.25
0.5 MgO-ZrO_2_	10	140.85	7.91	130.39	153.26
1 MgO-ZrO_2_	10	152.85	19.03	130.58	198.45

The incorporation of MgO and ZrO₂ NPs, alone or in combination, resulted in considerable improvements in the surface and strength properties of the heat-cured resin, as shown in Table 6. The most significant synergistic effect was observed with the 1 wt.% MgO‒ZrO₂ group, which yielded the greatest improvement in flexural strength (+67.7%) and a reduction in surface roughness (−42.9%), whereas 0.5% MgO‒ZrO_2_ resulted in statistically significant increases in density (+0.72) and hardness (+7.2). The increases in flexural strength and surface roughness could be the most clinically relevant, even with the minor rise and decline in density and hardness respectively was observed at the higher concentration. Therefore, the ultralow concentrations of the NPs have very minor effects of the density and hardness. The main mechanical failure mode of the denture bases may be directly addressed by strength improvement, which leads to a considerable decrease in the frequency of fractures. Similarly, a decrease in surface roughness could be effective to lower the adherence of bacteria, which thus might decrease the risk of denture stomatitis while improving patient oral hygiene.

**Table 6:** Mean percentage improvement in the properties of heat-cured acrylic samples containing different amounts of nanoparticles relative to the control.

Groups	Density improvement (%)	Surface roughness improvement (%)	Vickers hardness improvement (%)	Flexural strength improvement (%)
Control	0.00	0.0	+0.0	0.00
0.5% MgO	+0.18	−16.0	+5.8	+27.4
1% MgO	+0.57	−24.2	−3.2	+44.4
0.5% ZrO_2_	+0.28	−12.4	+7.1	+14.4
1% ZrO_2_	+0.51	−27.5	−1.8	+32.5
0.5% MgO-ZrO_2_	+0.72	−13.3	+7.2	+54.5
1% MgO-ZrO_2_	+0.51	−42.9	−3.7	+67.7

### 3.7 Fracture analysis

**Figure 10:**
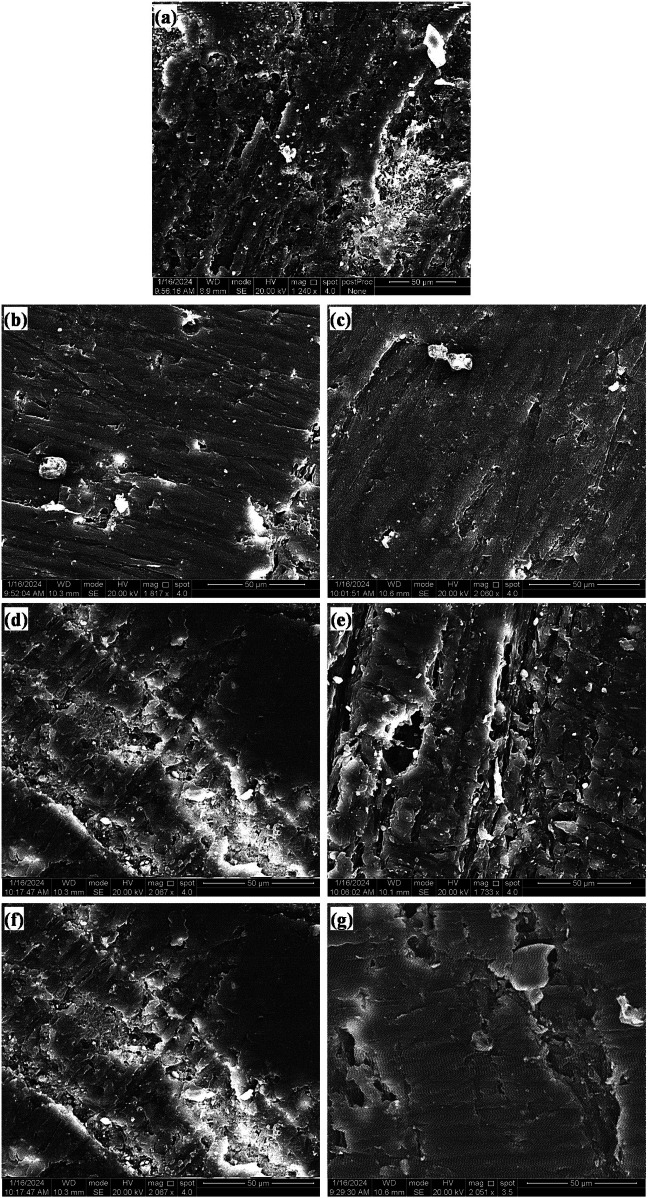
SEM images of the fracture topography of acrylic resin samples of the **(a)** control group and those with **(b)** 0.5 MgO, **(c)** 1.0 MgO, **(d)** 0.5 ZrO_2_, **(e)** 1.0 ZrO_2_, **(f)** 0.5 MgO‒ZrO_2_, and **(g)** 1.0 MgO‒ZrO_2_.

Fractographic examination of the fractured surfaces of the heat-cured acrylic resin samples verified that all the samples could have fragments A and B perfectly and smoothly fit back together with no visible fracture line. The fracture outlines were comparable in all of the test groups and were classified as brittle fractures, as shown in Figure 10.

## 4 Discussion

For decades, dental practitioners have relied on PMMA to manufacture durable and biocompatible denture bases to rehabilitate both the aesthetic appearance and functional chewing capacity of patients who have lost their natural teeth (38). Nevertheless, the physical and mechanical characteristics of these materials are not ideal. Their low resistance to impact and bending stress, for example, often leads to cracks or breaks during handling (25). The integration of NPs into denture base materials has been widely researched, but their effects on material properties continue to be a subject of debate (26, 33, 39). While certain studies have proposed enhancements in strength, biocompatibility and other key characteristics, others have raised concerns about potential drawbacks or inconsistencies. As a result, the use of NPs in denture bases remains a complex and evolving area of study. The incorporation of additive particles such as ZrO₂ and MgO into PMMA materials has been proposed individually in certain studies, although their synergistic effects on structural changes, strength, and performance have not been fully explored. Therefore, our novel study aimed to explore how adding MgO and ZrO₂ NPs at various concentrations either individually or together affects the polymerization process, density, surface roughness, hardness and flexural strength of heat-cured acrylic resin denture base materials. This study supported our hypothesis, as the addition of MgO and ZrO₂ NPs remarkably altered the chemical environment of the acrylic polymer. The additives worked in collaboration to increase the density and flexural strength while decreasing the surface roughness and slightly lowering the hardness at elevated nanoparticle concentrations.

In the literature, different approaches have been proposed for integrating NPs into denture resins, including direct mixing, *in situ* polymerization, surface modification, ultrasonication, hybrid reinforcement, and grafting with nanotubes or nanofibers (40). Direct mixing, either manually or via centrifugal force, is commonly used due to its ease and compatibility with NPs, though *in situ* polymerization stimulates consistent nanoparticle dispersion during PMMA synthesis, as established by calcium carbonate NPs. Surface modification with coupling agents and salinized fillers improves NPs interfacial adhesion and dispersion with the possibility of cytotoxic effect due to their degradation. On the other hand, agglomerates are efficiently reduced by ultrasonication to create a uniform mixture (40).

Among these methods, direct mixing and ultrasonication were selected for this study because of their affordability and ease of implementation This technique been found effective in achieving uniform NPs spreading without compromising mechanical properties (8). No salinization or coupling agents used in the present study because we aimed to test the direct effects of the each nanomaterial itself without possible contribution of residual treatment agents and cytotoxicity concern. The results of the EDX analysis and TEM images confirmed the existence and good dispersion of these NPs within the heat–cured resin samples, indicating that our mixing methods effectively reduced the aggregation of the NPs.

To assess the chemical and structural properties of the produced nanocomposite material, FTIR analysis was used to study the influence of the NPs. Polymerization of the developed composite resin. The results showed that adding MgO and ZrO₂ NPs changed the chemical environment of the acrylic polymer by improving the interaction with the polymer chains. Interestingly, the combination of MgO and ZrO₂ had a synergistic effect, indicating stronger interactions with the polymer chains. This interaction likely contributes to the observed improvements in strength, as the NPs may facilitate a more compact and homogenous resin structure. The pure acrylic spectrum showed distinct peaks at 1,626 ± 2.1 cm^−^^1^, corresponding to the C = O stretch of the ester carbonyl group, along with C-H stretching vibrations between 2,950 ± 1.2 and 2,850 ± 1.7 cm^−^^1^ and C-O stretching vibrations in the 1,150 ± 1.8 and 1,250 ± 1.5 cm^−^^1^ range. These findings align with the known structure of acrylic polymers, as previously reported (41, 42).

When ZrO_2_ is added, especially at a higher concentration of 1.0%, the C = O stretching peak shifts slightly to 1,735 ± 1.9 cm^−^^1^. This shift suggests that the electronic environment around the carbonyl group changes due to the coordination bonding between Zr and the oxygen atoms in the ester groups (43). New peaks ranging from 1,600 ± 2.5 to 1,675 ± 2.2 cm^−^^1^ appear, which are attributed to Zr–O bonds, further confirming the chemical interaction of Zr with the acrylic matrix, which is in line with the results of research by Reyes–Acosta et al. (44).

In parallel, the addition of Mg led to a wide and stronger shift of the C = O peak to 1,739 ± 1.7 cm^−^^1^ (SD, *n* = 3), in addition to the release of a new band peak at 1,631 ± 1.1 cm^−^^1^ (SD, *n* = 3), assigned to Mg‒O vibration bonds, indicating the potential for stronger ionic or coordination interactions than those of Zr (45). XPS analysis of Al_2_O_3_, MgO and ZrO_2_ confirmed Al-O, Mg-O and Zr-O bonds inferred from FTIR shifts (46, 47). However, the FTIR spectrum of the Zr and Mg incorporated into the acrylic material shows a synergistic effect via the C = O peak shifting further to 1,732 ± 1.8 cm^−^^1^ and the simultaneous presence of both Zr-O (1,628 ± 2.9 cm^−^^1^) and Mg-O (1,650 ± 1.7 cm^−^^1^) peaks (48). These observations indicate that Zr and Mg interact essentially with carbonyl oxygen atoms, which in turn leads to the formation of coordination and ionic bonds and polymer confinement effects within the polymer matrix (47).

These interactions are expected to enhance the thermal stability and mechanical properties of the material because the C–H stretching intensity decreases, the C–O region changes, and chain mobility and potential structural modifications are restricted (49). Similarly, the filler particles used in the present study were MgO (20–30 nm) and ZrO₂ (30 ± 5 nm), which were smaller than the resin particles (121.2 μm); in turn, it was anticipated that they would fill the gaps between the polymer particles and build a uniform mixture without causing any disruptions to the segments of the polymer chain (37, 50).

The current study revealed that adding MgO and ZrO₂ NPs to acrylic resin increased their density, regardless of their type and weight percentage. This improvement is likely due to the nanofillers filling interstitial spaces within the polymer structure, reducing voids and improving structural integrity. In general, higher-density materials can reduce the number of pores or microgaps throughout processing, which is a frequent challenge with acrylic resin prostheses (37). The porosity of the polymer tolerates dynamic fluid movement while providing active sites for the sequestration of molecules (37, 51). In this study, we used MgO and ZrO₂ NPs, which were previously identified to be insoluble in water (52, 53). These NPs serve as crosslinkers, which increase the cross-link density and decrease the total volume of the matrix of susceptible polymers. Our results revealed that the NPs in the treated groups were denser than those in the untreated pure control. The highest increase was 1.1859 g/cm^3^ in the 0.5 MgO-ZrO_2_-treated group compared to the lowest value 1.1774 g/cm^3^ in the control group. This improvement can be attributed to a “packing effect” and the crystalline nature of both the MgO and ZrO_2_ NPs, where they fill interstitial spaces, leading to a more compact resin structure and reduced porosity (35, 54). The findings of the present study are consistent with those of Dos Santos et al. (2023), who reported high density values for PMMA modified with cerium dioxide (CeO_2_), silicon dioxide (SiO_2_), and titanium dioxide NPs (37). However, our study uniquely demonstrated that combined MgO-ZrO₂ incorporation yields a more significant effect than either nanoparticle alone does. While the absolute alterations at approximately 0.7% densification is small, this reflects a positive indicator of fundamental changes in the denture resin, such as a less porous structure with improved mechanical properties resulting from the loading of ZrO₂ and MgO. Thus, the clinical value of these materials does not change in mass but rather strengthens the materials for better durability and patient comfort.

Similarly, surface roughness decreased significantly in the nanoparticle-modified groups, which represents a critical advancement in the development of high-performance denture base materials. The rough surface and porosity of dental restorations tend to result in plaque accumulation, bacterial adhesion, increased staining susceptibility, and faster degradation over time (7). Our results reveal that the combination of MgO and ZrO₂ NPs meaningfully enhances surface smoothness, with the dual-phase 1% MgO‒ZrO₂ composite reaching the most refined surface structure. This improvement is consistent with a clear dose‒dependent association, wherein increased nanoparticle loading correlates with progressive surface refinement, a trend that highlights the potential for altering material properties through defined compositional control. The smoothing effect may result from the NPs acting as microscopic fillers dispersed within the resin matrix during the polymerization process. These particles occupy interfacial voids and defects, effectively reducing surface irregularities and micro-porosities while enhancing structural homogeneity. These results align with findings reported by Alnamel and Mudhaffer when low-concentration silica (SiO₂) NPs were incorporated with acrylic resin materials (55). The primary reason for improved surface roughness could be the filling effect by the NPs. However, some variability might come from uncontrolled inconsistency in polishing.

The analogous presentation of MgO and ZrO₂ as individual additives suggests that both the NPs exert similar surface-modifying mechanisms within the acrylic resin matrix. This inidicates that surface-smoothing effects are mostly concentration dependent rather than material specific. This phenomenon may be attributed to their shared role as nanofillers, which efficiently occupy microvoids and irregularities during the polymerization process. The NPs likely act as nucleation sites, promoting a more homogeneous resin microstructure and reducing the formation of surface defects. Notably, the synergistic effect observed in the MgO‒ZrO₂ hybrid group implies that the combination of these NPs introduces complementary interactions, potentially through differences in particle morphology, surface energy, or interfacial bonding, that amplify their individual benefits. For example, the high hardness of ZrO₂ (32, 56) and antimicrobial properties of MgO (57) may collectively enhance both mechanical and functional outcomes, although further research to validate this theory is needed.

The dose-dependent refinement observation aligns with research on polymer nanocomposites, where optimum nanoparticle stuffing balances distribution efficiency and agglomeration risks (55). Our results suggest that at a 1% concentration, the MgO‒ZrO₂ hybrid achieves a critical threshold of nanoparticle distribution with lower clustering, which could otherwise compromise surface integrity. This contrasts with prior studies reporting increased roughness at higher filler concentrations due to aggregation, highlighting the importance of nanoparticle compatibility and dispersion techniques in acrylic resins (58).

The increase in the Vickers hardness of the heat-cured acrylic resin incorporated with the lowest concentration of 0.5% MgO and ZrO₂ NPs, either individually or in combination, demonstrated logical expectations in the nanocomposite strategy, where nanoparticle fillers are classically engaged to enhance the strength properties. However, our findings reveal an adverse relationship between nanoparticle concentration and surface hardness. This may reflect a critical threshold of NPs beyond which agglomeration overtakes dispersion. These observations are consistent with other studies reporting a reverse relationship between nanoparticle concentration and surface hardness, where higher NPs loadings resulted in diminished performance (7). The highest mechanical properties of resins reported in previous studies were achieved with 0.1% TiO_2_ NPs (59). Using higher concentrations may encourage agglomeration, generating localized stress points and disrupting the material's homogeneity (7).

The most prominent improvement was observed in the acrylic resin containing 0.5% MgO-ZrO_2_ NPs. This suggests a synergistic interaction between MgO and ZrO_2_ NPs at lower concentrations, potentially enhancing the structural integrity of the acrylic resin matrix by filling microvoids and reinforcing interfacial bonding. This mechanism aligns with earlier studies highlighting the role of NPs in improving polymer hardness through dispersion and interfacial adhesion (60, 61). These findings have practical implications for the design of denture bases, where surface hardness is essential for wear resistance and durability (7).

Another important finding of the current study is the substantial improvement in the flexural strength of heat-cured acrylic resin reformed with MgO and ZrO₂ NPs, either individually or in combination, in a dose-dependent manner. This progressive improvement might be attributed to the inherent mechanical properties of the ceramic NPs. The integration of NPs, particularly at higher concentrations, effectively reinforces the resin matrix by modifying inherent structural properties, such as porosity and microcracks. The greater performance of the 1% MgO-ZrO₂ group may stem from synergistic interactions between the two nanoparticle types. Magnesium oxide NPs, known for their high stiffness (22), which can increase the fracture toughness of ZrO₂, collectively increase the ability of the resulting resin to resist bending forces without catastrophic failure (62). NPs may reinforce the ability of resin to tolerate bending forces, possibly through crack deflection mechanisms and improved stress distribution (22). A greater surface area of nanoscale particles has been shown to increase the interfacial interactions of dental resin and improve its mechanical properties (58).

The concentration-dependent increase in flexural strength departs from the Vickers hardness findings, where higher concentrations of MgO and ZrO_2,_ either alone or together, reduced hardness, most likely as a result of agglomeration. This variance suggests that flexural strength and surface hardness are governed by different reinforcing mechanisms. Even though clustering NPs at higher quantities can create localized defects that weaken the surface, the material becomes better at resisting bending forces because those NPs strengthen its bulk structure. Even in the case of partial accumulation, a larger number of NPs are expected to generate an even more consistent matrix for stress distribution, increasing resistance to bending forces. In a previous research by Gad et al. (2016), single fillers of nano-ZrO_2_ at different concentrations (2.5 wt.%, 5 wt.%, and 7.5 wt.%) were used to repair the denture base. The authors reported that these particles increased the flexural strength of the repaired parts but decreased the impact strength, especially at high nano-ZrO_2_ concentrations (27).

The brittle fracture patterns observed in all the samples were characterized by clean, smooth fractures without fragmentation, indicating a uniform stress distribution across the modified resin. Compared with previous studies, our observations align with the inherent brittleness of conventional heat-cured acrylic resins, which are prone to sudden failure under stress. In particular, the addition of NPs significantly improved the flexural strength and did not alter the fundamental brittle nature of the material. This suggests that the NPs primarily enhance resistance to crack initiation rather than propagation, reinforcing the matrix without imparting ductility (63). Similar findings have been reported in nanocomposites, where fillers improve strength but do not necessarily transition fracture modes from brittle to ductile (64).

Our findings demonstrate that the integration of MgO and ZrO₂ NPs prompts dissimilar changes in the physicochemical structure of acrylic resin matrices, which is consistent with the proposed hypothesis. Additionally, the synergistic interplay of the nano additives increased the bulk density and flexural stiffness while diminishing surface imperfections. However, at higher nanoparticle concentrations, there was a decrease in hardness, which was most likely due to localized dispersion effects. In comparison with other studies that integrated multiple NPs with PMMA, one recent research stated a detrimental effect on wear volume with increasing MgO NPs concentration in a composite of PMMA-5ZrO₂, the wear volume increased from 1.55 mm^3^ to 5.64 mm^3^ as the MgO additive increased from 2% to 6%. This effect was attributed to the agglomeration of MgO particles (25). The present results confirmed that these ultralow-dose hybrid nanocomposites obtained via direct mixing and ultrasonication are promising candidates to achieve a multiproperty enhancement in the flexural strength and surface finish without compromising density or hardness while higher NPs loadings often improve one property over the others.

One limitation of this study is the optimal dose for clinical durability under thermocycling, fatigue testing or long-term water sorption analysis and *in vivo* validation. Moreover, the biocompatibility, cytotoxicity and long-term degradation of the of the developed composite resin over time were not examined here. Thus, these factors should be considered in future studies. Hybrid formulations that balance both properties while optimizing dispersion methods (e.g., surface modification) to minimize agglomeration at higher concentrations should also be explored.

This study shows clinically significant progress in the development of dental materials, demonstrating that PMMA denture-based resin reinforced with MgO-ZrO₂ NPs can improve patient outcomes by improving strength properties and surface finish, which in turn can minimize plaque adherence, microbial colonization, and mechanical wear in denture bases and orthodontic devices, offering a pathway to optimize patient-specific devices with enhanced mechanical integrity and reduced biofilm colonization. However, challenges such as stability throughout time and the high cost of some NPs must be addressed before their widespread acceptance. Clinical trials are essential to validate real-world performance and market viability.

## 5 Conclusion

This study revealed that MgO and ZrO₂ NPs, either independently or in combination, significantly modified the properties of heat-cured acrylic resin used for denture bases. The synergistic effect of the MgO and ZrO₂ NPs at low concentrations (0.5–1.0 wt.%) enhanced the density and flexural strength while reducing the surface roughness of the resulted nanocomposites.

## Data Availability

The original contributions presented in the study are included in the article/Supplementary Material, further inquiries can be directed to the corresponding author.

## Appendix

**Table A1:** Descriptive statistics for the density, surface roughness, vickers hardness and flexural strength of heat-cured acrylic resin incorporated with 0.5% MgO, (C) 1% MgO, 0.5% ZrO_2_, 1% ZrO_2_, 0.5% MgO-ZrO_2_, 1% MgO-ZrO_2_ and the control.

Tukey's multiple comparisons test	Density	Surface roughness	Vickers hardness	Flexural strength
Control vs. 0.5% MgO	0.043	0.005	0.031	0.000
Control vs. 1% MgO	0.000	0.000	0.240	0.000
Control vs. 0.5% ZrO_2_	0.002	0.027	0.009	0.008
Control vs. 1% ZrO_2_	0.000	0.000	0.511	0.000
Control vs. 0.5% MgO-ZrO_2_	0.000	0.020	0.008	0.000
Control vs. 1% MgO-ZrO_2_	0.000	0.000	0.167	0.000
0.5% MgO vs. 1% MgO	0.000	0.132	0.001	0.002
0.5% MgO vs. 0.5%ZrO_2_	0.248	0.536	0.637	0.017
0.5% MgO vs. 1%ZrO_2_	0.000	0.037	0.005	0.334
0.5% MgO vs. 0.5% MgO-ZrO_2_	0.000	0.622	0.608	0.000
0.5% MgO vs. 1% MgO-ZrO_2_	0.000	0.000	0.000	0.000
1% MgO vs. 0.5%ZrO_2_	0.001	0.035	0.000	0.000
1% MgO vs. 1%ZrO_2_	0.511	0.547	0.601	0.028
1% MgO vs. 0.5% MgO-ZrO_2_	0.082	0.047	0.000	0.059
1% MgO vs. 1% MgO-ZrO_2_	0.457	0.001	0.833	0.000
0.5% ZrO_2_ vs. 1% ZrO_2_	0.010	0.007	0.001	0.001
0.5% ZrO_2_ vs. 0.5% MgO-ZrO_2_	0.000	0.899	0.966	0.000
0.5% ZrO_2_ vs. 1% MgO-ZrO_2_	0.013	0.000	0.000	0.000
1% ZrO_2_ vs. 0.5% MgO-ZrO_2_	0.018	0.010	0.001	0.000
1% ZrO_2_ vs. 1% MgO-ZrO_2_	0.930	0.027	0.465	0.000
0.5% MgO-ZrO_2_ vs. 1% MgO-ZrO_2_	0.014	0.020	0.000	0.015
